# Supplementary material for: Predictability of HOMA-IR for Gestational Diabetes Mellitus in Early Pregnancy Based on Different First Trimester BMI Values
Source: J Pers Med. 2022 Dec 28;13(1):60. doi: 10.3390/jpm13010060 (PMC9866419; doi:10.3390/jpm13010060)
Supplement: Supplementary file 1 [file jpm-13-00060-s001.zip › jpm-2105314-supplementary.pdf]

Supplemental Table S1. Laboratory data and pregnancy outcomes in participants divided by FT-BMI.

	Group 1: FT-BMI <24 kg/m^2 (n = 1066)	Group 2: 24 kg/m^2 ≤FT-BMI <28 kg/m^2 (n = 210)	Group 3: FT-BMI ≥28 kg/m^2 (n = 67)	P-value, 1 vs 2	*p*-value, 1 vs 3	*p*-value, 2 vs 3
			Characteristics in the third trimester of pregnancy			
Incidence of GDM	191 ((17.92%)	73 ((34.76%)	36 ((53.73%)	**<0.001****	**<0.001****	**0.006****
Family history of diabetes	148 (13.88%)	37 (17.62%)	10 (14.93%)	0.160	0.811	0.610
GWG (kg)	13.0 (11.0, 16.0)	12.0 ((9.0, 15.0)	9.5 ((3.9, 12.1)	**<0.001****	**<0.001****	**<0.001****
SBP (mmHg)	117.0 ((110.0, 120.0)	120.0 ((110.0, 123.0)	117.5 ((111.0, 120.5)	**<0.001****	0.874	0.089
DBP ((mmHg)	72.0 ((70.0, 80.0)	72.5 ((70.0, 80.0)	70.0 ((70.0, 80.00)	0.084	0.516	0.156
Preterm delivery	25 ((2.82%)	15 ((8.67%)	4 ((7.41%)	**<0.001****	0.058	0.770
			Newborn birth weight (kg)			
<2500 g	24 ((2.71%)	9 ((5.20%)	3 ((5.56%)			
>2500 g, <4000 g	841 ((94.81%)	154 ((89.02%)	47 ((87.04%)	**0.013***	**0.044***	0.903
≥4000 g	22 ((2.48%)	10 ((5.78%)	4 ((7.41%)			
			Laboratory data between 6–12 weeks of pregnancy			
FBG ((mmol/L)	4.5 ((4.2, 4.9)	4.7 ((4.3, 5.1)	4.45 ((4.1, 5.0)	**0.007****	0.630	0.085
FCP ((ng/ml)	0.79 ((0.61, 1.01)	1.05 ((0.74, 1.37)	1.53 ((1.15, 1.98)	**<0.001****	**<0.001****	**<0.001****
FI ((uU/ml)	6.0 ((4.3, 8.1)	8.2 ((6.5, 11.3)	11.0 ((9.3, 15.4)	**<0.001****	**<0.001****	**<0.001****
HOMA-IR	1.22 ((0.86, 1.68)	1.70 ((1.33, 2.39)	2.35 ((1.73, 3.11)	**<0.001****	**<0.001****	**0.001****
HOMA-β	120.00 ((75.36, 192.00)	142.00 ((97.50, 237.83)	235.55 ((157.06, 391.25)	**<0.001****	**<0.001****	**<0.001****
QUICKI	0.37 ((0.35, 0.39)	0.35 ((0.33, 0.37)	0.34 ((0.32, 0.35)	**<0.001****	**<0.001****	**0.001****
TC ((mmol/L)	4.00 ((3.50, 4.53)	4.20 ((3.70, 4.77)	4.31 ((3.81, 4.74)	**<0.001****	**0.002****	0.521
TG ((mmol/L)	0.84 ((0.65, 1.08)	0.95 ((0.69, 1.36)	1.11 ((0.80, 1.56)	**<0.001****	**<0.001****	0.189
HDL-C (mmol/L)	1.50 ((1.29, 1.70)	1.38 ((1.19, 1.63)	1.34 ((1.18, 1.50)	**0.002****	**0.001****	0.215
LDL-C (mmol/L)	1.98 ((1.66, 2.38)	2.28 ((1.86, 2.69)	2.48 ((2.04, 2.87)	**<0.001****	**<0.001****	**0.044***

* $p<0.05$; ** $p<0.01$.

Supplemental Table S2. Baseline characteristics and laboratory data in participants divided by FT-BMI and GDM.

Characteristics		Group 1: Normal weight (FT-BMI <24.0 kg/m^2) with NGT	Group 2: Normal weight (FT-BMI <24.0 kg/m^2) with GDM	Group 3: Overweight (FT-BMI ≥24.0 kg/m^2) with NGT	Group 4: Overweight (FT-BMI ≥24.0 kg/m^2) with GDM	*p*-value
Total		875 ((65.15%)	191 ((14.22%)	168 ((12.51%)	109 ((8.12%)	
Maternal baseline information						
Age (years)		29.0 (27.0, 32.0)	30.0 (28.0, 33.0)	30.0 (27.0, 33.0)	32.0 (30.0, 35.0)	**<0.001****
Gravidity	1	477 ((54.51%)	106 ((55.50%)	87 ((51.79%)	50 ((45.87%)	0.730
	2	234 ((26.74%)	55 ((28.80%)	49 ((29.17%)	29 ((26.61%)	
	≥3	164 ((18.74%)	30 ((15.71%)	32 ((19.05%)	30 ((27.52%)	
Parity	0	626 ((71.54%)	139 ((72.77%)	117 ((69.64%)	63 ((57.80%)	0.073
	1	235 ((26.86%)	47 ((24.61%)	49 ((29.17%)	43 ((39.45%)	
	≥2	14 ((1.60%)	5 ((2.62%)	3 ((1.79%)	3 ((2.75%)	
Family history of diabetes		119 ((13.60%)	29 ((15.18%)	21 ((12.50%)	26 ((23.85%)	**0.031***
History of adverse pregnancy [a]		142 ((16.23%)	41 ((21.47%)	34 ((20.24%)	25 ((22.94%)	0.123
Laboratory data between 6–12 weeks of pregnancy						
FBG ((mmol/L)		4.5 (4.2, 4.9)	4.8 (4.4, 5.3)	4.6 (4.2, 4.9)	4.9 (4.4, 5.5)	**<0.001****
FCP ((ng/ml)		0.76 ((0.59, 0.98)	0.93 ((0.71, 1.14)	1.03 ((0.75, 1.38)	1.33 ((0.95, 1.96)	**<0.001****
FI ((uU/ml)		5.60 ((4.10, 7.60)	7.20 ((5.20, 9.70)	7.80 ((6.00, 10.45)	12.30 ((8.30, 16.80)	**<0.001****
HOMA-IR		1.15 ((0.80, 1.55)	1.50 ((1.07, 2.15)	1.62 ((1.20, 2.21)	2.51 ((1.76, 3.82)	**<0.001****
HOMA-β		120.00 ((72.90, 192.25)	112.00 ((77.62, 171.67)	150.00 ((102.31, 235.83)	170.00 ((106.67, 330.00)	**<0.001****
QUICKI		0.37 ((0.36, 0.40)	0.36 ((0.34, 0.38)	0.36 ((0.34, 0.37)	0.33 ((0.31, 0.35)	**<0.001****
TC ((mmol/L)		3.92 ((3.46, 4.48)	3.98 ((3.52, 4.54)	4.20 ((3.72, 4.57)	4.30 ((3.70, 4.87)	**<0.001****
TG ((mmol/L)		0.82 ((0.64, 1.05)	0.91 ((0.72, 1.22)	0.87 ((0.67, 1.29)	1.17 ((0.81, 1.56)	**<0.001****
HDL-C ((mmol/L)		1.50 ((1.30, 1.70)	1.46 ((1.25, 1.61)	1.38 ((1.19, 1.63)	1.37 ((1.18, 1.57)	**<0.001****
LDL-C ((mmol/L)		1.95 ((1.63, 2.34)	2.04 ((1.68, 2.53)	2.27 ((1.86, 2.59)	2.37 ((1.93, 2.94)	**<0.001****

a: Defined as embryo damage, spontaneous abortion or preterm delivery in previous pregnancy. Data are presented as n (%) or median (interquartile range). * $p<0.05$; ** $p<0.01$. The *p* value shown in the table was among the four subgroups.
